# Initial measurement of ion nonextensive parameter with geodesic acoustic mode theory

**DOI:** 10.1038/s41598-022-07295-0

**Published:** 2022-03-01

**Authors:** Huibin Qiu, Donghua Xiao, Junjie Wu, Shengfa Wu, Chengjie Zhong, Xiaobin Li, Xingkun Peng, Youlong Yuan, Qilong Cai, Jinming Chang, Tianyi Hu, Zuozhi Hu, Yuqing Zhu

**Affiliations:** 1 Jiangxi Province Key Laboratory of Fusion and Information Control, Department of Physics, Nanchang University, Nanchang, 330031 China; 2 NCU-ASIPP Magnetic Confinement Fusion Joint Lab, Institute of Fusion Energy and Plasma Application, Nanchang University, Nanchang, 330031 China

**Keywords:** Plasma physics, Magnetically confined plasmas

## Abstract

The consideration of nonextensivity effects is crucial to the accurate diagnosis of plasma parameters; common plasma nonextensive parameters include electron nonextensive parameter and ion nonextensive parameter, and the former can be measured, while the ion nonextensive parameter cannot be measured yet. Here we show the measurement of ion nonextensive parameter of plasma based on the theory of nonextensive geodesic acoustic modes. We assume that the plasma to be measured can be described by nonextensive statistical mechanics, and on this basis, the nonextensive geodesic acoustic mode theory is established. Utilizing this theory, we have measured the ion nonextensive parameter ${{q}_{{{F}_{\mathrm {i}}}}}= 1.565$ which cannot be diagnosed even by a nonextensive single electric probe. Our research points out that the proposed measurement method of ion nonextensive parameter may play a role in plasma diagnosis and will help us to grasp the nonextensivity of plasma more precisely. We hope the proposed method of ion nonextensive parameter diagnosis based on the nonextensive geodesic acoustic mode theory can be the starting point of more complex ion nonextensive parameter diagnosis methods. In addition, the measurement of ion nonextensive parameter is closely related to the study of various plasma waves, instabilities, turbulence and abnormal transport, and a defined and quantitative test of nonextensive geodesic acoustic mode theory will bound up deeply with such developments.

## Introduction

Theoretical analysis and a large number of experiments prove that the components of the plasma do not satisfy Boltzmann–Gibbs statistics and can be well described by nonextensive statistical mechanics^1–3^. The consideration of nonextensivity effects is very important to the accurate diagnosis of plasma parameters. When the nonextensivity effects are not considered, the diagnosis error of the electric probe may be as high as 83.91%^4^. Therefore, we must consider the influence of nonextensive parameters and the measurement of the nonextensive parameters is a must. Common plasma nonextensive parameters include electron nonextensive parameter and ion nonextensive parameter. We have been able to measure electron nonextensive parameter^1,5^. However, the ion nonextensive parameter cannot be measured yet. Here we show the measurement of ion nonextensive parameter of plasma based on the theory of nonextensive geodesic acoustic modes. We assume that the plasma to be measured can be described by nonextensive statistical mechanics, and on this basis, establish the nonextensive geodesic acoustic mode theory^6^. Using this theory, we have measured the ion nonextensive parameter of 1.565 which cannot be measured even by a nonextensive single electric probe^1^. Our research shows that the proposed measurement method of ion nonextensive parameter may play a role in plasma diagnosis and will help us to grasp the nonextensivity of plasma more precisely. We hope the proposed method of ion nonextensive parameter diagnosis based on the nonextensive geodesic acoustic mode theory can be the starting point of more complex ion nonextensive parameter diagnosis methods. For example, it is possible to develop an ion nonextensive parameter diagnosis based on nonextensive electric probe which includes effects of elongation, triangle deformation, electron and so on. In addition, the measurement of ion nonextensive parameter is closely related to the study of various plasma waves, instabilities, turbulence and abnormal transport. A defined and quantitative test of nonextensive geodesic acoustic mode theory^6^ will be relevant for such developments.

**Figure 1:**
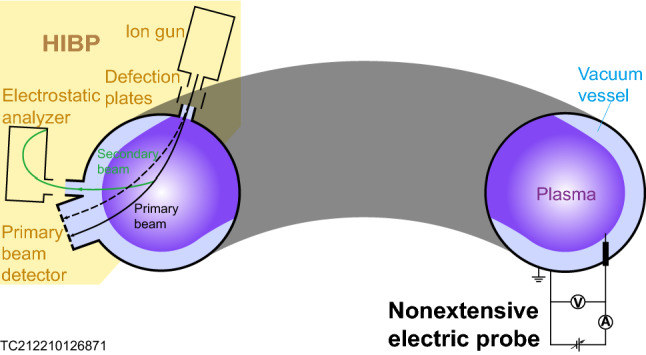
Schematic of ion nonextensive parameter diagnosis method based on nonextensive geodesic acoustic mode theory.

**Figure 2:**
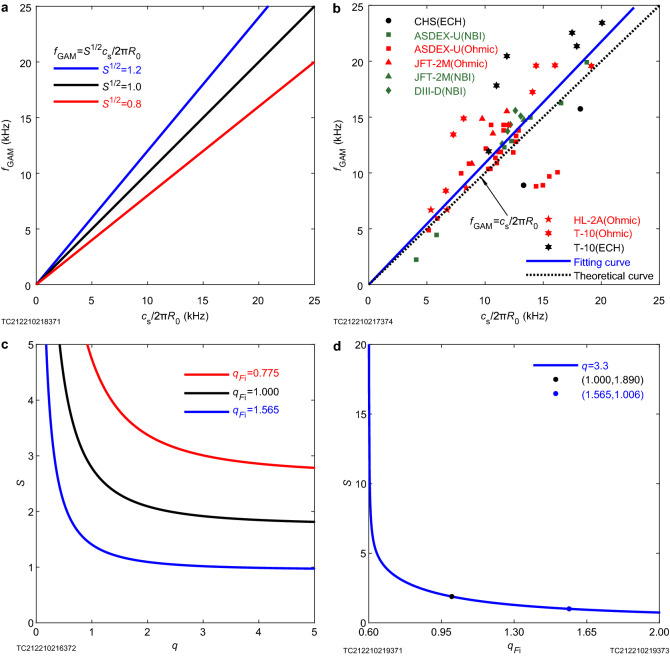
Analysis diagram of nonextensive geodesic acoustic mode law. (**a**) The theory of nonextensive geodesic acoustic mode shows that: the frequency of geodesic acoustic mode is directly proportional to ion sound velocity, but the proportional coefficient depends on the safety factor and ion nonextensive parameters. At the extensive limit, the relevant conclusions return to the results under the Boltzmann–Gibbs statistical framework. (**b**) The analysis of 58 experimental data points of 6 major devices also supports the conclusion that the geodesic acoustic mode frequency is proportional to the ion sound velocity. (**c**) The variation curve of *S* in the geodesic acoustic mode scaling law with the safety factor *q* shows that the geodesic acoustic mode frequency decreases as the safety factor *q* increases when the ion sound velocity ${{c}_{\mathrm{s}}}$ and the large radius ${{R}_{0}}$ are constant. (**d**) The variation curve of *S* in the geodesic acoustic mode scaling law with the ion nonextensive parameter ${ {q}_{{ { F}_{\mathrm{i}}}}}$ indicates that as the ion nonextensive parameter decreases, the geodesic acoustic mode frequency gradually increases (for details see the information of figure given in the “Methods”).

The ion nonextensive parameter diagnosis method based on nonextensive geodesic acoustic mode theory is a method that adopts the nonextensive geodesic acoustic mode theory^6^ as a foundation and combines the measurement of geodesic acoustic mode frequency and plasma electron temperature to give ion nonextensive parameter (Fig. 1). It can be used to diagnose the ion nonextensive parameter of high-temperature plasma in the boundary area of the tokamak device or in the divertor, which is a precondition for studying plasma waves, instability, turbulence and abnormal transport. Nowadays, more and more evidences show that nonextensive statistical mechanics can be considered as the basis of a more suitable theoretical framework to describe complex systems whose properties cannot be described by Boltzmann–Gibbs statistical mechanics^2,7^. Recent plasma diagnostic research^1^ shows that if nonextensive statistical mechanics is selected to describe the plasma, it will not only cover the results under the Boltzmann–Gibbs statistical mechanics framework, at the same time prove the correctness of the theory itself at the extensive limit, but also has the advantage of being able to draw conclusions which can cover at least three other situations. And after evaluating with a set of real measurement data, it is found that if the nonextensivity effect of plasma is not considered, the diagnosis error can be as high as $\text {83.91}\%$, which shows that the actual measurement should consider the influence of nonextensive parameters^4^. Usually nonextensive parameters include electron nonextensive parameters and ion nonextensive parameters. However, currently only electron nonextensive parameters can be diagnosed by the newly invented nonextensive single electric probe^1,5^, while ion nonextensive parameters cannot be diagnosed yet. Here, we put forward an ion nonextensive parameter diagnosis method based on the nonextensive geodesic acoustic mode theory through combining the existing diagnostic methods of geodesic acoustic mode frequency and plasma electron temperature, such as nonextensive single electric probe^1^, with nonextensive geodesic acoustic mode theory, which can measure the ion nonextensive parameter that cannot be measured even with a nonextensive single electric probe^1^. We assume that the plasma to be measured can be described by nonextensive statistical mechanics, and on this basis, establish the nonextensive geodesic acoustic mode theory. Using this theory, we have measured the ion nonextensive parameter of $\text {1.565}$ which cannot be measured even by a nonextensive single electric probe.

## Nonextensive geodesic acoustic mode theory

In order to obtain the geodesic acoustic mode theory under the nonextensive statistical framework consistent with the experiment, we extend the geodesic acoustic mode theory under the Boltzmann–Gibbs statistical framework to the theory under the nonextensive statistical framework. The obtained geodesic acoustic mode dispersion relationship under the nonextensive statistical framework is as follows (see “Methods”):

1 $$\begin{aligned} {{f}_{\mathrm{GAM}}}=\frac{{{\omega }_{\mathrm{GAM}}}}{\text{2 }\uppi }=\sqrt{S\left( {{q}_{{{F}_{\mathrm{i}}}}},q \right) }\frac{{{c}_{\mathrm{s}}}}{\text{2 }\uppi {{R}_{0}}}, \end{aligned}$$

where

2 $$\begin{aligned} S\left( {{q}_{{{F}_{\text {i}}}}},q \right) =\frac{\text{7 }}{\text {4}\left( \text{3 }{{q}_{{{F}_{\text {i}}}}}\text {-}\text{1 } \right) }\left\{ \text{1 }+\sqrt{\text{1 }+\frac{\text{4 }}{{{q}^{\text{2 }}}}\frac{\frac{\text {23}}{\text{2 }\left( \text{5 }{{q}_{{{F}_{\text {i}}}}}\text {-}\text{3 } \right) \left( \text{3 }{{q}_{{{F}_{\text {i}}}}}\text {-}\text{1 } \right) }}{{{\left[ \text {-}\frac{\text{7 }}{\text{2 }\left( \text{3 }{{q}_{{{F}_{\text {i}}}}}\text {-}\text{1 } \right) } \right] }^{\text{2 }}}}} \right\} , \end{aligned}$$

when $q_{F_{\mathrm {i}}}>\frac{\text{1 }}{\text{3 }}$. The above formula is clearly illustrated in Fig. 2 as follows: it can be seen from Fig. 2a that the geodesic acoustic mode frequency is proportional to the ion sound velocity, which is supported by the fluid^8^ and kinetic theory^9^ and experimental data (Fig. 2b), while what is different from the theory under the Boltzmann–Gibbs (extensive) statistical framework is that the proportional coefficient is not only a function of the safety factor, but also a function of nonextensive parameters: the proportional coefficient decreases with the increase of the safety factor (Fig. 2c), and also decreases with the increase of the ion nonextensive parameter (Fig. 2d). This indicates that the geodesic acoustic mode ${{f}_{\mathrm{GAM}}}\text {--}{{ {c}_{\mathrm{s}}}}/{\mathrm{2}\uppi { {R}_\mathrm{0}}}$ curve (the theoretical cornerstone of ion nonextensive parameter diagnosis) has a kind of complicated dependence on nonextensive parameters, which is different from the traditional (excluding nonextensive parameters) geodesic acoustic mode theory; in addition, we found that at the extensive limit (${{q}_{{{F}_{\mathrm {i}}}}}=\text{1 }$), the above results all return to the traditional theory based on Boltzmann–Gibbs statistical framework, which supports the correctness and universality of the nonextensive theory (namely a larger scope of application).

**Table 1:** Parameters related to plasma generated by 36815 shot on T-10 Tokamak device.

$\sqrt{{{{\textsf {\textit{S}}}}}}$	${{{\textsf {\textit{SSE}}}}}$	${{\textsf {\textit{R}}}}^{{\textsf {\textit{2}}}}$	${{{\textsf {\textit{q}}}}}$	${{{\textsf {\textit{q}}}}}_{{{{\textsf {\textit{F}}}}}_{{{\textsf {\tt{i}}}}}}$
1.003	0.284	0.992	3.3	1.565

**Figure 3:**
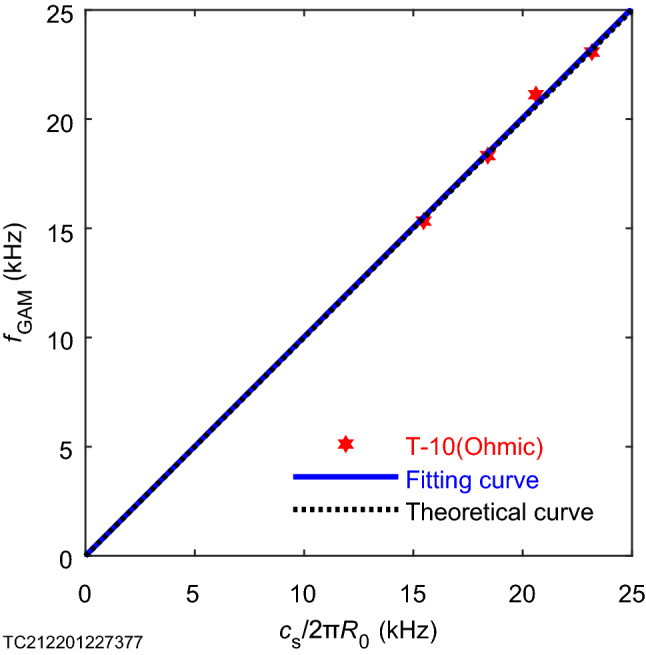
Analysis diagram of geodesic acoustic mode experimental data on T-10 device. An analysis of 4 experimental data obtained from 36815 shot on T-10 device using the HIBP and 2nd ECE harmonic methods^10^ shows that the slope of the curve obtained by no-intercept linear fitting was 1.003, as shown in Table 1 (for details see the information of figure given in “Methods”).

The above analysis has shown that nonextensive parameters have an influence on the geodesic acoustic mode ${{f}_{\mathrm{GAM}}}\text {--}{{ c _{\mathrm{s}}}}/{\mathrm{2}\uppi { R _{0}}}$ curve; based on this theory, next, we will explain how to measure ion nonextensive parameters that cannot be measured even with a nonextensive single electric probe^1,5^.

## Ion nonextensive parameter measurement

**Figure 4:**
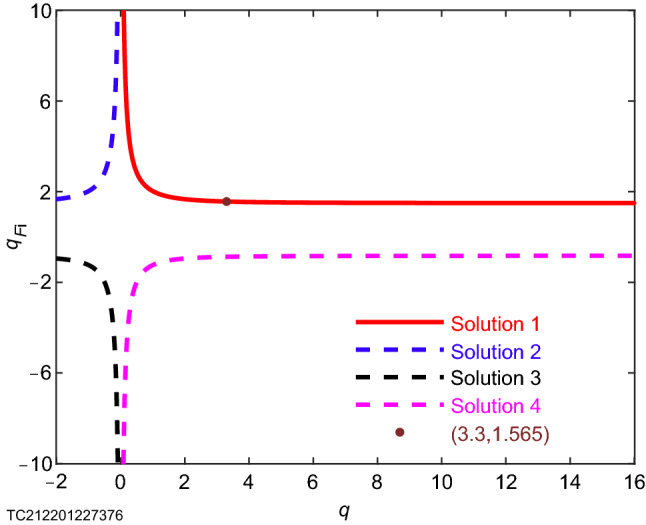
Measurement diagram of ion nonextensive parameter of plasma generated by 36815 shot on T-10 device. The four curves are the ${{q}_{{{F}_{\text {i}}}}}-{q}$ figure given by Eq. (2) combining with the slope $\sqrt{{S}}={1.003}$ (Table 1) obtained from Fig. 3. As you can see from the diagram, there are four groups of solutions, and here, from a physical point of view, we only consider the group of solutions represented by the red real curve. ${{q}_{{{F}_{\mathrm {i}}}}}={1.565}$ is the ion nonextensive parameter of plasma generated by 36815 shot (safety factor^11^ ${q}={3.3}$) on T-10 device.

With the purpose of measuring ion nonextensive parameters, we can first measure a set of geodesic acoustic mode frequencies and electron temperature for the plasma to be measured in a specific tokamak device through existing methods (such as the frequency measurement methods HIBP^10^ used on T-10 device and nonextensive electric probes^1^, Fig. 1). In this work, we obtain 4 experimental data points for the plasma generated by 36815 shot of the T-10 device (Fig. 3). Then the least square method is used to fit this set of experimental data. Since both the theory^7,8^ and the experiment (Fig. 2b) have proved the relationship between ${{f}_{\mathrm{GAM}}}$ and ${{c}_{\mathrm{s}}}/\text{2 }{\uppi }{{R}_\mathrm{{0}}}$ is the direct proportional, we make a linear fitting of the direct proportional function without intercept. It turns out that the optimal slope is$\sqrt{S}=\text {1.003}$ (Table 1). Then, according to Eq. (2), the relationship between the safety factor *q* and the ion nonextensive parameter ${{q}_{{{F}_{\mathrm{i}}}}}$ is known (Fig. 4). Since $q=\text {3.3}$ is the safety factor of 36815 shot on T-10 device^11^, the corresponding ion nonextensive parameter ${{q}_{{{F}_{\mathrm{i}}}}}=\text {1.565}$ can be solved.

**Figure 5:**
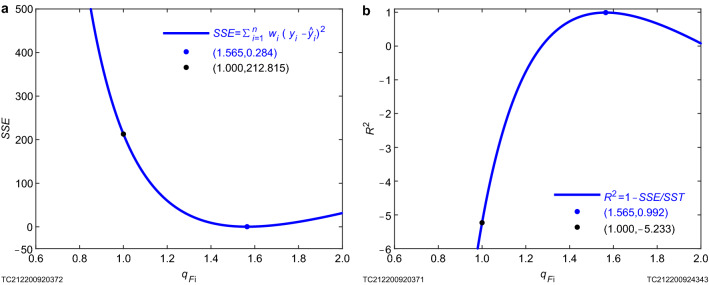
Analysis graphs of statistics *SSE* and ${R}^{\text{2 }}$ for the optimal ion nonextensive parameter ${{{q}_{{{F}_{\text {i}}}}}=\text {1.565}}$. For details, see the information of figure given in “Methods”.

In order to illustrate that ${{q}_{{{F}_{\mathrm{i}}}}}=\text {1.565}$ is the optimal ion nonextensive parameter, statistics *SSE* and ${{R}^{\text{2 }}}$ are analyzed. We found that when the ion nonextensive parameter ${{q}_{{{F}_{\mathrm{i}}}}}$ takes different values, *SSE* also takes different values (Fig. 5a) and gets the minimum value when ${{q}_{{{F}_{\mathrm{i}}}}}=\text {1.565}$, indicating that ${{q}_{{{F}_{\mathrm{i}}}}}=\text {1.565}$ is the optimal ion nonextensive parameter. In order to confirm the reliability of the results measured with the statistic *SSE*, we also analyzed another independent indicator ${{R}^{\text{2 }}}$, and made an ${{R}^{\text{2 }}}$-${{q}_{{{F}_{\mathrm{i}}}}}$ graph, and also found that when ${{q}_{{{F}_{\mathrm{i}}}}}=\text {1.565}$, ${{R}^{\text{2 }}}$ achieves the maximum value (Fig. 5b), which confirms ${{q}_{{{F}_{\mathrm{i}}}}}=\text {1.565}$ is the optimal ion nonextensive parameter, that is, the measurement result of the ion nonextensive parameter of the plasma generated by 36815 shot on T-10 tokamak device is ${{q}_{{{F}_{\mathrm{i}}}}}=\text {1.565}$ (Table 1).

## Discussion and conclusion

Our research results show the effectiveness of a method for measuring ion nonextensive parameters in a tokamak device. Recent studies^1^ have shown that replacing Boltzmann–Gibbs statistical mechanics with nonextensive statistical mechanics has a strong advantage in describing plasmas. The diagnosis error without using nonextensive statistical mechanics to describe the plasma may be as high as $\text {83.91}\%$^4^. We established the theory of nonextensive geodesic acoustic mode by introducing nonextensive statistical mechanics to take into account the system nonextensivity which has been proven by a large number of facts^3,12–31^. This theory not only can obtain the related results^8,9^ of the traditional geodesic acoustic mode at the extensive limit, which proves the correctness of the nonextensive geodesic acoustic mode theory, but also can measure the ion nonextensive parameter ($\text {1.565}$, Fig. 4 and Table 1) that cannot be measured even with a nonextensive single electric probe^1^ by combining the existing diagnosis methods of geodesic acoustic mode frequency and plasma electron temperature (such as HIBP^10^ and nonextensive electric probe^1^, Fig. 1).

Our work fills the gap where the electron nonextensive parameter can be measured with the nonextensive single electric probe, but the corresponding ion nonextensive parameter cannot be diagnosed yet in the field of nonextensive parameters diagnosis.

Our research is the starting point of ion nonextensive parameter diagnosis. The methods of ion nonextensive parameter diagnosis which include effects of plasma elongation, triangle deformation or electron are being solved.

## Methods

Here, we use the standard model magnetic field^32^

3 $$\begin{aligned} {\varvec{B}}={{B}_{0}}\left\{ \left[ {1}/{\left( 1+\varepsilon \cos \theta \right) {{{\varvec{e}}}_{\phi }}} \right] +\left( {\varepsilon }/{q}\right) {{{\varvec{e}}}_{\theta }} \right\} , \end{aligned}$$

to consider a simple axisymmetric toroidal system, where $\phi$ and $\theta$ are the toroidal and poloidal angles, respectively, and *q* represents the safety factor here, and it is assumed that the inverse aspect ratio $\varepsilon =a/{{R}_{0}}$ is relatively small. Assume that the electrostatic potential on the magnetic surface $a={{a}_{0}}$ is strictly constant. This simplification works for ${{T}_{\text {e}}}\ll {{T}_{\text {i}}}$, because the poloidal change of the potential energy is related to a finite ${{{T}_{\text {e}}}}/{{{T}_{\text {i}}}}$^33^. Consider the electrostatic potential $\varphi =\sum \nolimits _{\omega ,k}{\hat{\varphi }\exp \left[ \text {i}k\left( a-{{a}_{0}} \right) -\text {i}\omega t \right] }$, and the *k* component of the perturbed distribution function ${\hat{f}}$ can be written as ${\hat{f}}=q\hat{\varphi }{\partial {{F}_{0}}}/{\partial E}+{\hat{h}}{{\text {J}}_{0}}\left( k\rho \right)$, where the energy of the particle is $E={m{\textit{v}\,^{2}}}/{2}$, and ${{F}_{0}}$ is selected to be the nonextensive distribution function^34^. ${{\text {J}}_{0}}$ is the Bessel function, $\rho ={{\textit{v}_{\bot }}}/{\varOmega }$ is the gyroradius, and ${\varOmega }$ is the gyrofrequency. ${\hat{h}}\left( {\textit{v}_{\parallel }},{\textit{v}_{\bot }},\theta \right)$ satisfies the linear gyrokinetic equation^35^:

4 $$\begin{aligned} \left( \omega -{{\omega }_{\text {d}}}\sin \theta +\text {i}{{\omega }_{\text {t}}}\frac{\partial }{\partial \theta } \right) {\hat{h}}=\frac{q{{{{\hat{F}}}}_{0}}}{T}\omega {{\text {J}}_{0}}\hat{\varphi }, \end{aligned}$$

where ${{\omega }_{\text {t}}}=\frac{{\textit{v}_{\parallel }}}{q{{R}_{0}}}$, ${{\omega }_{\text {d}}}=k\left[ \frac{\left( 2v\,_{\parallel }^{2}+v\,_{\bot }^{2} \right) }{2{{R}_{0}}{\varOmega }} \right]$, ${{{\hat{F}}}_{0}}=\left( 2-{{q}_{{{F}_{\text {i}}}}} \right) \frac{{{n}_{0}}{{A}_{{{q}_{{{F}_{\text {i}}}}}}}}{{{\uppi }^{3/2}}v\,_{\text {ti}}^{3}}{{\left[ 1-\left( {{q}_{{{F}_{\text {i}}}}}-1 \right) \frac{{{\varvec{v}}^{2}}}{v\,_{\text {ti}}^{2}} \right] }^{\frac{2-{{q}_{{{F}_{\text {i}}}}}}{{{q}_{{{F}_{\text {i}}}}}-1}-1}}$. The linear gyrokinetic equation has the following analytical solution:

5 $$\begin{aligned} \begin{array}{cc} {\hat{h}}=\frac{q{{{{\hat{F}}}}_{0}}\omega {{\text {J}}_{0}}\hat{\varphi }}{T}\left\{ \begin{array}{l} \sum \limits _{m,n=-\infty }^{+\infty }{{{\text {i}}^{m-n}}{{\text {J}}_{n}}\left( \!\frac{{{\omega }_{\text {d}}}}{{{\omega }_{\text {t}}}} \right) {\text {J}}_{m}\left( \frac{{{\omega }_{\text {d}}}}{{{\omega }_{\text {t}}}} \right) \frac{\exp \left[ \text {i}\left( m-n \right) \theta \right] }{\omega +n{{\omega }_{\text {t}}}}}\end{array}\right\} . \end{array} \end{aligned}$$

Condition ${{v}_{\parallel }}\rightarrow \infty$, ${\hat{h}}\rightarrow 0$, Bessel function expansion $\exp \!\left( -\text {i}\frac{{{\omega }_{\text {d}}}}{{{\omega }_{\text {t}}}}\cos \theta \right) \!\!=\!\!\sum \limits _{n=-\infty }^{+\infty }\!\!\!{{{\left( -\text {i} \right) }^{n}}}{{\text {J}}_{n}}\left( \frac{{{\omega }_{\text {d}}}}{{{\omega }_{\text {t}}}} \right) \exp \left( -\text {i}n\theta \right)$, and periodic boundary conditions ${\hat{h}}\left( \theta \right) ={\hat{h}}\left( \theta +2\uppi \right)$ are used here. The subscript representing the particle type is hidden above. The ion response greatly controls the zonal flow dynamics, because when $k{{\rho }_{\text {i}}}\ll 1$, the electron response to potential $\varphi \left( a\right)$ is relatively small.

Under quasi-neutral condition, the governing equation of geodesic acoustic mode is given:

6 $$\begin{aligned} \int {{{R}_{0}}\text {d}\theta {{\text {d}}^{3}}{} \textit{v}}\left( {{\textit{v}}_{\text {d}}}{\hat{f}}\sin \theta -{{\textit{v}}_{\text {p}}}{{{{\hat{F}}}}_{0}} \right) =0, \end{aligned}$$

where ${{v}_{\text {d}}}={{{\omega }_{\text {d}}}}/{k}$ is curvature drift velocity, ${{v}_{\text {p}}}=g\omega k\hat{\varphi }/\left( \varOmega B \right)$ is polarization drift velocity, $g\approx 1-{{k}^{2}}\rho _{\text {i}}^{2}/4$ represents the finite gyroradius effect on the polarization current in an inhomogeneous field. This condition can be equivalently written in another form^8,9,33^: $\nabla \!\cdot \!{{{{\varvec{j}}}}}\sim \left\langle {\,{j}_{r}} \right\rangle =0$, where ${{{\varvec{j}}}}$ is perturbed current (including current caused by curvature drift and polarization current), ${{j}_{r}}$ is its radial component. Note that there is a trivial solution $\omega \equiv {{\omega }_{\text {r}}}+\text {i}\gamma =0$, which corresponds to the static zonal flow in analysis of Rosenbluth-Hinton at collisionless limit^36^. Here, we are looking for a non-trivial solution of $\left| \omega \right| \ne 0$. Under the assumption of large aspect ratio, only the term $\sin \theta$ contributes to the curvature drift current among the components given by Eq. (5), so Eq. (6) is rewritten as

7 $$\begin{aligned} \int \limits _{L}{\frac{{{\text {d}}^{3}}\textit{v}}{{{\uppi }^{3/2}}}\!\left( 2\!-\!{{q}_{{{F}_{\text {i}}}}} \right) {{A}_{{{q}_{{{F}_{\text {i}}}}}}}{{\left[ 1\!-\!\left( {{q}_{{{F}_{\text {i}}}}}\!-\!1 \right) {{\textit{v}}\,^{2}} \right] }^{\frac{2-{{q}_{{{F}_{\text {i}}}}}}{{{q}_{{{F}_{\text {i}}}}}-1}-1}}}\text {J}_{0}^{2}\left( k{{\textit{v}}_{\bot }} \right) \!\!\sum \limits _{n=0}^{+\infty }\!\!{\text {J}_{n}^{2}\!\left( \!\!kq\frac{2\textit{v}\,_{\parallel }^{2}+\textit{v}\,_{\bot }^{2}}{2{{\textit{v}}_{\parallel }}}\!\!\right) }\!\!\left( \!\frac{{{\textit{v}}_{\parallel }}}{\zeta /n-{{\textit{v}}_{\parallel }}}\!\!+\!\!\frac{-{{\textit{v}}_{\parallel }}}{\zeta /n+{{\textit{v}}_{\parallel }}}\!\right) =g\frac{{{k}^{2}}}{2}, \end{aligned}$$

where $\int _{L}^{{}}{{}}$ is the Landau route integral, and the following normalization and definition are used: $v= v/{{v}_{\mathrm {ti}}}$, $k=k{{v}_{\mathrm {ti}}}/{\varOmega }$ and $\zeta =q{{R}_{0}}\omega /{{v}_{\mathrm {ti}}}$, where ${{v}_{\mathrm {ti}}}=\sqrt{2{{\kappa }_{\text {B}}}{{T}_{\text {i}}}/{{m}_{\text {i}}}}$. The above equation describes the dynamics of geodesic acoustic modes in large aspect ratio circular geometry in which the plasma is nonextensive distributed and collisionless but the trapped particle effect is not considered. As we know, trapped particles play a very important role in the long-time behavior of zonal flow, namely residual flow or stationary zonal flow^36^. However, due to the low bounce frequency $\sqrt{2\varepsilon }{{v}_{\text {ti}}}/q{{R}_{0}}$ of trapped particles, especially in the limit of large aspect ratio, it is expected that trapped particles will not be involved in the resonance process of geodesic acoustic mode oscillation in large quantities. Because it is difficult for the trapped ion to maintain parallel resonance velocity and repeat bouncing motion at the same time, even at low parallel phase velocity, the trapped ion is not as effective as the passing ion^37^. The finite-gyroradius effect enhances the landau damping of the electrostatic perturbations and makes it effective, even at $\left| \omega \right| \gg \left| {{k}_{\parallel }}{{v}_{\text {ti}}} \right|$^38^, so we expect the finite *k* enhanced geodesic acoustic mode damping, which has been confirmed by Ref.^37^. Here, we will focus on the case where $k\rightarrow 0$, but $k\hat{\varphi }$ is finite, which reduces to drift-kinetic model. The above equation reduces to

8 $$\begin{aligned} G\left( \zeta \right) \equiv \frac{1}{{{q}^{2}}}+\frac{\left( -q_{{{F}_{\text {i}}}}^{2}+2{{q}_{{{F}_{\text {i}}}}}+1 \right) }{2{{q}_{{{F}_{\text {i}}}}}}\left[ \frac{1}{2}+{{\zeta }^{2}}\frac{1+{{q}_{{{F}_{\text {i}}}}}}{2}+{{\zeta }^{3}}{{Z}_{{{q}_{{{F}_{\text {i}}}}}}}\left( \zeta \right) \right] +\frac{1}{{{q}_{{{F}_{\text {i}}}}}}\left[ \frac{1+{{q}_{{{F}_{\text {i}}}}}}{2}+\zeta {{Z}_{{{q}_{{{F}_{\text {i}}}}}}}\left( \zeta \right) \right] +\frac{{{Z}_{{{q}_{{{F}_{\text {i}}}}}}}\left( \zeta \right) }{2\zeta {{q}_{{{F}_{\text {i}}}}}}=0, \end{aligned}$$

where

9 $$\begin{aligned} {{Z}_{{{q}_{{{F}_{\text {i}}}}}}}\!\left( \zeta \right) \equiv {{\text { }\uppi \text { }}^{-1/2}}\!\!\int \limits _{-{{\zeta }_{\max }}}^{{{\zeta }_{\max }}}{\frac{{{A}_{{{q}_{{{F}_{\text {i}}}}}}}{{\left[ 1-\left( {{q}_{{{F}_{\text {i}}}}}-1 \right) {{t}^{2}} \right] }^{\frac{1}{{{q}_{{{F}_{\text {i}}}}}-1}-1}}}{t-\zeta }}\text {d}t, \end{aligned}$$

is the *q*-modified plasma dispersion function, and ${{\zeta }_{\max }}=+\infty$ when $-1<{{q}_{{{F}_{\text {i}}}}}\le 1$; ${{\zeta }_{\max }}=1/\sqrt{{{q}_{{{F}_{\text {i}}}}}-1}$ when ${{q}_{{{F}_{\text {i}}}}}>1$. When $\left| \zeta \right| \gg 1$ and $\left| {{\zeta }_{\text {r}}} \right| \gg \left| {{\zeta }_{\text {i}}} \right|$, the plasma dispersion function can be made the large argument expansion, and the following formula is given

10 $$\begin{aligned} {{Z}_{{{q}_{{{F}_{\text {i}}}}}}}\!\left( \zeta \right) \!=\!\text {i}{{\uppi }^{1/2}}{{A}_{{{q}_{{{F}_{\text {i}}}}}}}{{\left[ 1\!\!-\!\left( {{q}_{{{F}_{\text {i}}}}}\!-\!\!1 \right) {{\zeta }^{2}}\right] }^{\frac{1}{{{q}_{{{F}_{\text {i}}}}}-1}-1}}-\!{{\zeta }^{-1}}\!\!\left[ \frac{1+{{q}_{{{F}_{\text {i}}}}}}{2}\!+\!\frac{1}{2{{\zeta }^{2}}}\!+\!\frac{3}{2\left( 3{{q}_{{{F}_{\text {i}}}}}-1 \right) {{\zeta }^{4}}}\!+\!\frac{15}{2\left( 5{{q}_{{{F}_{\text {i}}}}}-3 \right) \left( 3{{q}_{{{F}_{\text {i}}}}}-1 \right) {{\zeta }^{6}}}\!+\!\ldots \right] , \end{aligned}$$

It is easy to see that in the limit ${{q}_{{{F}_{\text {i}}}}}\rightarrow 1$, the above formula returns to the large argument expansion of Maxwellian plasma dispersion function^39^

11 $$\begin{aligned} Z\!\left( \zeta \right) \!\!=\!\!\text {i}{{\uppi }^{1/2}}{{\text {e}}^{-{{\zeta 
}^{2}}}}\!-\!{{\zeta }^{-1}}\!\!\left[ 1+\frac{1}{2{{\zeta }^{2}}}+\frac{3}{4{{\zeta }^{4}}}+\frac{15}{8{{\zeta }^{6}}}+\ldots \right] . \end{aligned}$$

Substituting the asymptotic expansion into Eq. (8) and retain the $O\left( {{\zeta }^{-4}} \right)$ terms, then

12 $$\begin{aligned}{{G}_\mathrm{a}}\left( \zeta \right) & \equiv \frac{1}{{{q}^{2}}} - \frac{7}{2\left( 3{{q}_{{{F}_{\text {i}}}}}-1 \right) {{\zeta }^{2}}} - \frac{23}{2\left( 5{{q}_{{{F}_{\text {i}}}}}-3 \right) \left( 3{{q}_{{{F}_{\text {i}}}}}-1 \right) {{\zeta }^{4}}}\\&\quad +\!\frac{\left( -q_{{{F}_{\text {i}}}}^{2}+2{{q}_{{{F}_{\text {i}}}}}+1 \right) }{2{{q}_{{{F}_{\text {i}}}}}}\text {i}{\sqrt{\uppi}} {{\zeta }^{3}}{{A}_{{{q}_{{{F}_{\text {i}}}}}}}{{\left[ 1 - \left( {{q}_{{{F}_{\text {i}}}}}-1 \right) {{\zeta }^{2}} \right] }^{\frac{1}{{{q}_{{{F}_{\text {i}}}}}-1}-1}}=0. \end{aligned}$$

At the limit ${{q}_{{{F}_{\text {i}}}}}\rightarrow 1$, Eq. (12) returns the result of the Maxwellian distributed plasma^9^:

13 $$\begin{aligned} {{G}_\mathrm{a}}\left( \zeta \right) \equiv \frac{1}{{{q}^{2}}}-\frac{7}{4{{\zeta }^{2}}}-\frac{23}{8{{\zeta }^{4}}}+\text{ i }\sqrt{\text { }\!\!\uppi \!\!\text { }}\sigma {{\zeta }^{3}}\exp \left( -{{\zeta }^{2}} \right) =0. \end{aligned}$$

Under the assumption of $\left| \gamma \right| \ll \left| {{\omega }_{\text {r}}} \right|$, Eq. (12) is reduced to two equations of real part and imaginary part, where the real part equation is

14 $$\begin{aligned} \frac{1}{{{q}^{2}}}-\left[ \frac{7}{2\left( 3{{q}_{{{F}_{\text {i}}}}}-1 \right) } \right] \frac{1}{{{\left( {q{{R}_{0}}{{\omega }_{\text {r}}}}/{{{\textit{v}}_{\text {ti}}}} \right) }^{2}}}-\left[ \frac{23}{2\left( 5{{q}_{{{F}_{\text {i}}}}}-3 \right) \left( 3{{q}_{{{F}_{\text {i}}}}}-1 \right) } \right] \frac{1}{{{\left( {q{{R}_{0}}{{\omega }_{\text {r}}}}/{{{\textit{v}}_{\text {ti}}}} \right) }^{4}}}=0. \end{aligned}$$

From Eq. (14), the real frequency part of the geodesic acoustic mode can be obtained as

15 $$\begin{aligned} {{\omega }_{\text {r}}}=\frac{{{\textit{v}}_{\text {ti}}}}{{{R}_{0}}}\sqrt{S\left( {{q}_{{{F}_{\text {i}}}}},q \right) }\simeq \frac{{{c}_{\text {s}}}}{{{R}_{0}}}\sqrt{S\left( {{q}_{{{F}_{\text {i}}}}},q \right) }, \end{aligned}$$

where

16 $$\begin{aligned} S\!\left( {{q}_{{{F}_{\text {i}}}}},q \right) \!\!=\!\!\frac{7}{\text {4}\left( 3{{q}_{{{F}_{\text {i}}}}}\!\!-\!\!1 \right) }\!\!\left\{ \!1\!+\!\!\!\!\sqrt{\!1\!\!+\!\!\frac{4}{{{q}^{2}}}\frac{\frac{23}{2\left( 5{{q}_{{{F}_{\text {i}}}}}-3 \right) \left( 3{{q}_{{{F}_{\text {i}}}}}-1 \right) }}{{{\left[ -\frac{7}{2\left( 3{{q}_{{{F}_{\text {i}}}}}-1 \right) } \right] }^{2}}}} \!\right\} , \end{aligned}$$

when $q_{F_{\text {i}}}>\frac{1}{3}$. When *q* is large, Eq. (15) becomes

17 $$\begin{aligned} \omega _{\text {GAM}}^{2}\!\!=\!\!\frac{7}{2\left( 3{{q}_{{{F}_{\text {i}}}}}-1 \right) }\frac{\textit{v}_{\text {ti}}^{2}}{R_{0}^{2}}\left[ 1+\frac{1}{{{q}^{2}}}\frac{46\left( 3{{q}_{{{F}_{\text {i}}}}}-1 \right) }{49\left( 5{{q}_{{{F}_{\text {i}}}}}-3 \right) } \right] ; \end{aligned}$$

and when $$q\rightarrow \infty$$,

18 $$\begin{aligned} {{\omega }_{\text {r}}}=\sqrt{\frac{7}{2\left( 3{{q}_{{{F}_{\text {i}}}}}-1 \right) }}\frac{{{\textit{v}}_{\text {ti}}}}{{{R}_{0}}}. \end{aligned}$$

When ${{q}_{{{F}_{\text {i}}}}}\rightarrow 1$, Eqs. (15), (17) and (18) all return to the results^9,40^ under the case of Maxwellian distribution.

Figure 2a is an analysis diagram of geodesic acoustic mode scaling law. The theoretical curve is given by derivation of plasma gyrokinetic, in which the black line returns to the scaling law given by fluid theory^8^, which supports the correctness of the gyrokinetic theory under the nonextensive statistical framework^6^. Analysis of 58 experimental data from 6 devices (including 2 from CHS device, 29 from ASDEX-U device, 5 from JFT-2M device, 5 from DIII-D device, 3 from HL-2A device and 14 from T-10 device^41^) also shows that fitting effect of the proportional function is better than the linear function with intercept (Fig. 2b). The coefficient of determination for proportional fitting is 0.95107, which is closer to 1 than the coefficient of determination for linear function fitting with intercept 0.55201, indicating that the effect of proportional fitting is better. The abscissa ${{{c}_{\text {s}}}}/{2\uppi {{R}_{0}}}$ is the reciprocal of the time for the sound wave to go around the large torus, where ${{R}_{0}}$ is the major radius of the tokamak. The experimental data on the T-10 device^10^ that this work focuses on is analyzed using the formula ${{c}_{\text {s}}}=\sqrt{{{{T}_{\text {e}}}}/{{{m}_{\text {i}}}}}$. The abscissa has a value range of (0, 25). On T-10 and TEXT tokamak, heavy ion beam probe (HIBP) diagnostic technology was used to study the specific oscillation of “20 kHz mode”^11^. The six devices (focus on the T-10 device) involved in this work have geodesic acoustic mode frequencies less than 25 kHz. The ordinate is the geodesic acoustic mode frequency, and the value range is (0, 25) kHz, because the ordinate and the abscissa have a proportional relationship with a slope close to 1 (Fig. 2b). On the T-10 device, Melnikov et al.^10^ used HIBP and multipin Langmuir probe to diagnose the scaling relations between the geodesic acoustic mode frequency and the electron temperature. The curve as a whole grows proportionally. The mathematical reason for this trend is that ${\text {d}{{f}_{\text {GAM}}}}/{\text {d}\left( {{{c}_{\text {s}}}}/{2\uppi {{R}_{\text {0}}}} \right) }$ is greater than zero. The geodesic acoustic mode is a unique electrostatic oscillation in the toroidal plasma. Its mode structure is symmetrical in the toroidal direction and approximately symmetrical in the polar direction. Its period is close to the time when the sound wave revolves around the large torus. The geodesic acoustic mode has a large radial electric field and is accompanied by a density perturbation with a polar modulus $m=1$^8^. The physical mechanism is the balance between drift and polarization drift caused by the geodesic component of the curvature of the magnetic field^9^. When the safety factor is fixed and the ion nonextensive parameter is greater than 0.6, the geodesic acoustic mode frequency decreases with the increase of the ion nonextensive parameter (see Figs. 2c and d for details).

Figure 2b is the diagram of experimental data analysis of geodesic acoustic mode on 6 large devices. There are 58 experiment data points, including 2 from CHS device, 29 from ASDEX-U device, 5 from JFT-2M device, 5 from DIII-D device, 3 from HL-2A device and 14 from T-10 device. The data acquisition method of CHS^42^, T-10^10^ and JFT-2M^43^ devices is heavy ion beam probe; the data acquisition methods of ASDEX-U^44^ device, DIII-D^45^ device and HL-2A^46^ device are Doppler reflector, beam emission spectroscopy and three step Langmuir probe arrays, respectively^41^. The theoretical curve is given by derivation of plasma gyrokinetic, which (black line) returns to the scaling law given by fluid theory, which proves the correctness of gyrokinetic theory under the framework of nonextensive statistics. The Ohmic means ohmic heating, which is the traditional heating mode of tokamak using transformer. Electron cyclotron heating (ECH) is the heating of electron cyclotron frequency range, and together with neutral beam injection (NBI) both are the auxiliary heating mode of tokamak device. The frequencies of the geodesic acoustic mode on the six devices in this work are less than 25 kHz. In this figure, ${{c}_{\text {s}}}$ is given indirectly by measuring the temperature. For example, the abscissas of the data points on the HL-2A device are indirectly given by measuring the electron temperature with the triple probe method^46^. The triple probe method is to indirectly derive the local plasma electron temperature by directly measuring the particle and energy flux flowing to the probe surface with solid conductor filaments. The geodesic acoustic mode frequency is proportional to ${{{c}_{\text {s}}}}/{2\uppi {{R}_{0}}}$, and the slope is of 1.088, we choose no intercept here. Because coefficient of determination (${{R}^{2}}$) of the curve obtained by no intercept fitting is 0.95107, and the curve coefficient of determination obtained by intercept fitting is 0.55201, and the closer this coefficient is to 1, the better the fitting effect is. In summary, the conclusion that the geodesic acoustic mode frequency is proportional to ${{{c}_{\text {s}}}}/{2\uppi {{R}_{0}}}$ is supported by plasma kinetics and fluid theory, and also by experimental data.

Figure 2c is the variation trend of *S* in geodesic acoustic mode scaling law with safety factor *q* under different ion nonextensive parameters. When the ion nonextensive parameter is ${{q}_{{{F}_{\text {i}}}}}=1.000$, the conclusion returns to result under the Boltzmann–Gibbs statistical framework. ${{q}_{{{F}_{\text {i}}}}}=1.565$ is an ion nonextensive parameter obtained by analyzing four experimental data of shot 36815 on T-10 device (Fig. 4). The abscissa *q* is the safety factor of the tokamak device. It is a parameter to describe the enclosed plasma magnetic surface, and it is also an important index of the device design and the operation of the plasma shot. The physical meaning is the number of circles in the direction of the large torus after a magnetic force line revolving around a small cross section. The calculation method^47^ is $q={{{{{B}_{\text {t}}}r}/{{{B}_{\text {p}}}R}}_{0}}$ ($B_{\mathrm {t}}$ is toroidal magnetic field; *r* is the distance between the magnetic surface of the magnetic field line and the magnetic axis; ${{B}_{\text {p}}}$ is magnetic field on plasma boundary in tokamak device, the calculation method of it is^47^ ${{B}_{\text {p}}}={{{\mu }_{0}}{{I}_{\text {p}}}}/{2{\uppi }a}$, *a* is minor radius of plasma, $I_{\mathrm {p}}$ is plasma current; $R_{\mathrm {0}}$ is the plasma major radius), the range of values is $[0, +\infty )$, in this work we choose [0, 5] , where $q=0$ means that the tokamak toroidal magnetic field is zero, and the reason why the safety factor greater than 5 is not selected is that the general range of safety factor of T-10 device is 2.5-4^10^. If $q<2.5$, the disruptions become more frequent and the confinement performance degrades relative to the scaling expression^48^. The ordinate is the coefficient *S* which depends on the safety factor and the ion nonextensive parameter in the scaling law ${{f}_{\text {GAM}}}=\sqrt{S}c{_{\text {s}}}/{2\uppi {{R}_{0}}}$ of geodesic acoustic mode. When ${{q}_{{{F}_{\text {i}}}}}=0.775$, the range of *S* is $\left[ 2.642, +\infty \right)$, and the *S* value range corresponding to the general range of safety factor of the T-10 device is $\left[ 2.858, 3.146\right]$; when $q_{F_{\mathrm {i}}}=1.000$, the value range of the *S* is $[1.750, +\infty )$, and the *S* value range corresponding to the general range of safety factor of the T-10 device is [1.847, 1.982]; when ${{q}_{{{F}_{\text {i}}}}}=1.\text {565}$, the value range of the *S* is $\left[ 0.947,\text { +}\infty \right)$ and the *S* value range corresponding to the general range of safety factor of the T-10 device is $\left[ 0.988, 1.046\right]$. It can be seen from the figure that when ${{q}_{{{F}_{\text {i}}}}}$ fixed, *S* decreases with the increase of *q*. The mathematical reason for this trend is that when ${{q}_{{{F}_{\text {i}}}}}$ is fixed, ${\text {d}S}/{\text {d}q}<0$. Combined with figure ${{f}_{\text {GAM}}}-{{{c}_{\text {s}}}}/{2\uppi {{R}_{0}}}$ (Fig. 2a), it can be seen that the geodesic acoustic mode frequency decreases with the increase of safety factor *q* when the ion sound velocity ${{c}_{\text {s}}}$ and major radius ${{R}_{0}}$ are fixed. It seems can be seen from the figure that when the safety factor *q* fixed, the curve decreases with the increase of the ion nonextensive parameter ${{q}_{{{F}_{\text {i}}}}}$, and the detailed analysis is shown in the $S-{{q}_{{{F}_{\text {i}}}}}$ diagram.

Figure 2d is the trend diagram of coefficient $S$ changes with ion nonextensive parameter $q_{F_\mathrm{i}}$ in geodesic acoustic mode scaling law when safety factor $q=3.3$. The abscissa $q_{F_\mathrm{i}}$ is the ion nonextensive parameter of the plasma in the tokamak device, and its value range is $(-1, +\infty )$. In this figure, only (0.6, 2.0] is selected, and the reason why $q_{F_\mathrm{i}}<3/5$ interval is not considered is the occurrence of complex number, while the reason why ${{q}_{{{F}_{\text {i}}}}}={3}/{5}$ is not considered is that the denominator is zero. In view of the ion nonextensive parameter of the plasma generated by shot 36815 on T-10 device we are concerned about is 1.565, in this work we focus on the interval ${{q}_{{{F}_{\text {i}}}}}\le \text {2}$. When ${{q}_{{{F}_{\text {i}}}}}={3}/{5}$, the ordinate takes $+\infty$; $q_{F_\mathrm{i}}=1$ is the extensive limit, and in this case, the results return to those under the Boltzmann–Gibbs statistical framework^34,49^; $q_{F_\mathrm{i}}=1.565$ is the ion nonextensive parameter obtained by analyzing 36815 shot data^10^ on T-10 device (Fig. 4). The ordinate is the coefficient in the geodesic acoustic mode scaling law $f_\mathrm{GAM}=\sqrt{S}c_{\text{ s }}/2\uppi {R_\mathrm{0}}$ that depends on the ion nonextensive parameter under the condition that the safety factor $q=3.3$ for 36815 shot^10^ of the T-10 device, and the value range is $(0, +\infty )$. When $q_{F_\mathrm{i}}=3/5$, the corresponding ordinate is $+\infty$; $q_{F_\mathrm{i}}=1$, the ordinate value is 1.890, which is close to 2 and is consistent with the extensive scaling law^8^ $f_\mathrm{GAM}=\sqrt{2}c_{\text{ s }}/2\uppi { R_\mathrm{0}}$; $q_{F_\mathrm{i}}=1.565$, the ordinate value is 1.006. It can be seen from the figure that in the $q_{F_\mathrm{i}}\in (3/5, 2]$ region, *S* decreases monotonically with the increase of the ion nonextensive parameter $q_{F_\mathrm{i}}$. The mathematical reason for this change trend is that $\mathrm{d}S/\mathrm{d}q_{F_\mathrm{i}}<0$. The coordinates of special points on the curve are $(3/5, +\infty )$, (1.000, 1.890), (1.565, 1.006) and (2, 0.741). In the $q_{F_\mathrm{i}}\in (3/5, 2]$ region, with the decrease of ion nonextensive parameter from 2 to 0.6, the plasma temperature increases gradually, the free energy contained in the plasma increases gradually, and the geodesic acoustic mode frequency also increases gradually.

Figure 3 is the analysis graphics of geodesic acoustic mode experiment data on T-10 device. There are 4 data points in the figure, all of which are data obtained from shot 36815 on the T-10 device^10^. The method of obtaining these data is the heavy ion beam probe (HIBP)^10^. The theoretical curve is derived from the fluid^8^ method, and the fitting curve is fitted by the least square method. The coefficient of determination $R^2$ of the fitting curve is 0.992 (Table I). The closer this coefficient is to 1, the better the fitting effect. Ohmic is ohmic heating, which is a heating method in which tokamak uses a transformer for heating. The abscissa has a value range of (0, 25) kHz, and its average value is 19.4 kHz. At 0 kHz, the frequency of the geodesic acoustic mode becomes a positive number. The geodesic acoustic mode frequency of the T-10 device is less than 25 kHz. The ordinate $f_\mathrm{GAM}$ is the geodesic acoustic mode frequency, and the value range is (0, 25) kHz. The ordinate is approximately proportional to the abscissa, namely $f_\mathrm{GAM}=c_\mathrm{s}/2\uppi {R_{0}}$. The average ordinate of all data points is 19.5 kHz. On the T-10 device, Melnikov et al.^10^ using HIBP and Langmuir probe diagnosed the scaling relationship between the geodesic acoustic mode frequency and the electron temperature. The geodesic acoustic mode frequency $f_\mathrm{GAM}$ is proportional to $c_\mathrm{s}/2\uppi {R_{0}}$, and theoretically^8^ the slope of the curve is 1. However, the slope of the curve obtained by choosing the non-intercept fitting method is 1.003. According to the results of data fitting, the coefficient of determination $(R^2)$ of the curve with intercept is 0.9922, and the $R^2$ of the curve obtained without intercept is 0.9917. Although it seems that the curve fitting effect with intercept seems to be a little better (the $R^2$ obtained by the two methods are not much different), we have theoretically proved that the curve of the geodesic acoustic mode frequency with respect to $c_\mathrm{s}/2{\uppi }R_{0}$ passes the origin. And when analyzing 58 experimental data of geodesic acoustic modes on 6 major devices, what we got is that the effect of non-intercept fitting is better (Fig. 2b), so in the case of one device and fewer data points, we allow for better intercept fitting. In order to be consistent with the theoretical results and the results obtained by multiple devices and multiple data, we still use non-intercept fitting to present this figure. Whether it is a theoretical curve or a fitting curve, the geodesic acoustic mode frequency always increases monotonically with the increase of $c_\mathrm{s}/2{\uppi }R_{0}$. The mathematical reason for this change trend is that the first derivative of the geodesic acoustic mode frequency with respect to $c_\mathrm{s}/2{\uppi }R_{0}$ is greater than zero in the domain. The physical meaning is that as the reciprocal of the time for the sound wave to circumnavigate the large torus increases (namely, the time for the sound wave to circle the large torus decreases), the frequency of the geodesic acoustic mode increases monotonically.

Figure 5a is the variation curve of sum of squares due to error (*SSE*) with the ion nonextensive parameter when the value of the ion nonextensive parameter is accurate to 0.001. $q_{F_\mathrm{i}}=1.565$ is the ion nonextensive parameter obtained by analyzing 36815 shot data on T-10 device^10^. The ordinate is the sum of squares due to error *SSE*^50^, and its value range is [0.284, 215412.553], where ${SSE}(q_{F_\mathrm{i}})=212.815$, that is, under the extensive limit, the sum of squares due to error obtained by using gyrokinetic based on nonextensive statistical mechanics and that obtained by using traditional gyrokinetic is the same, which proves that the proposed nonextensive gyrokinetic is correct at the extensive limit. When $q_{F_\mathrm{i}}\rightarrow 0.6^{+}$, there is a maximum value ${SSE}(q_{F_\mathrm{i}}\rightarrow 0.6^{+})=215412.553$; when $q_{F_\mathrm{i}}=1.565$, there is a minimum value ${SSE}(q_{F_\mathrm{i}}=1.565)=0.284$; when $q_{F_\mathrm{i}}=2$, ${SSE}(q_{F_\mathrm{i}}=2)=31.510$. The curve of the sum of squares due to error that changes with ion nonextensive parameters obtained by using gyrokinetic based on nonextensive statistical mechanics first decreases and then increases, that is, the nonextensive fitting effect first becomes better and then becomes worse. Specifically, when $q_{F_\mathrm{i}}\rightarrow 0.6^{+}$, the maximum sum of squares due to error is ${SSE}(q_{F_\mathrm{i}}\rightarrow 0.6^{+})=215412.553$; when $q_{F_\mathrm{i}}\in (0.6, 1.0)$ with the increase of ion nonextensive parameter, the sum of squares due to error becomes smaller and smaller, but it is larger than that obtained by traditional gyrokinetic, and it is the same as that obtained by traditional gyrokinetic until $q_{F_\mathrm{i}}=1$, because at this time the nonextensive statistical mechanics is reduced to Boltzmann–Gibbs statistical mechanics, that is, the same theory is adopted at this time, and these two sums of squares due to error are naturally the same; when $q_{F_\mathrm{i}}\in (1.0, 1.565)$, the sum of squares due to error obtained by using gyrokinetic based on nonextensive statistical mechanics still decreases with the increase of the ion nonextensive parameter, until $q_{F_\mathrm{i}}=1.565$, the sum of squares due to error reaches the minimum value of 0.284, that is to say the fitting result is closest to reality at this time; when $q_{F_\mathrm{i}}\in (1.565, 2]$, the sum of squares due to error increases with the increase of ion nonextensive parameter, but it is still smaller than that obtained by traditional gyrokinetic. The mathematical reason for this change trend is that the sum of squares due to error has been optimized since the ion nonextensive parameter $q_{F_\mathrm{i}}$ increases from 0.6, and the optimal value of ${SSE}=0.284$ is obtained at $q_{F_\mathrm{i}}=1.565$, and after reaching the optimal value, the nonlinear fitting result becomes worse with the increase of nonextensive parameter; the physical reason for this change trend is that Boltzmann–Gibbs statistical mechanics is not an optimal statistical mechanics to describe the plasma system, but the nonextensive statistical mechanics can be adjusted to better describe the real plasma system because it has a nonextensive parameter; in this work, the real plasma system is described by the nonextensive statistical mechanics with an ion nonextensive parameter of 1.565.

Figure 5b is variation curve of coefficient of determination $R^2$ with the ion nonextensive parameter when the value of the ion nonextensive parameter is accurate to 0.001. $q_{F_\mathrm{i}}=1.565$ is the ion nonextensive parameter obtained by analyzing 4 experimental data^10^ of 36815 shot on T-10 device (Fig. 4), and it is consistent with the value obtained by using *SSE* as the goodness of fit (Fig. 5a), which confirms the correctness of the optimal value $q_{F_\mathrm{i}}=1.565$. The ordinate $R^2$ is the coefficient of determination, which is expressed by formula $R^{2}=1-\textit{SSE}/\textit{SST}$ (Fig. 5b) and can be used to judge the goodness of fit. Its value range is $(-\infty , 0.992)$. When $q_{F_\mathrm{i}}=0.6$, $R^2$ is $-\infty$; when $q_{F_\mathrm{i}}=1$, $R^2$ is $-5.233$; when $q_{F_\mathrm{i}}=1.565$, $R^2$ gets the maximum value 0.992; when $q_{F_\mathrm{i}}=2$, $R^2$ takes the value 0.077. The curve of the coefficient of determination $R^2$ obtained based on the theory of nonextensive statistical mechanics increases first and then decreases as the ion nonextensive parameter increase; there are 4 special point coordinates: $(3/5, -\infty )$, $(1.000, -5.233)$, (1.565, 0.992) and (2, 0.077). These 4 points divide the determination coefficient curve into 3 sections. When $q_{F_\mathrm{i}}\in (3/5, 1)$, $R^2$ obtains the minimum value $-\infty$ at $q_{F_\mathrm{i}}\rightarrow 3/5$, it increases monotonically with the increase of the nonextensive parameter, until it reaches $R^2(q_{F_\mathrm{i}}=1.000)=-5.233$ at $q_{F_\mathrm{i}}=1.000$; when $q_{F_\mathrm{i}}\in (1, 1.565)$, $R^2$ still follows the nonextensive parameter increases and monotonically increases until $q_{F_\mathrm{i}}=1.565$ reaches the maximum value $R^2(q_{F_\mathrm{i}}=1.565)=0.992$; when $q_{F_\mathrm{i}}\in (1.565, 2)$, $R^2$ decreases monotonously with the increase of the nonextensive parameter, that is, the value of $R^2$ afterwards is all less than $R^2(q_{F_\mathrm{i}}=1.565)=0.992$, and $R^2$ takes the value 0.077 until $q_{F_\mathrm{i}}=2$. The mathematical reason for this change trend is that the coefficient of determination increases first and then decreases as the ion nonextensive parameter $q_{F_\mathrm{i}}$ increases from 3/5 to 2. There is an inflection point $q_{F_\mathrm{i}}=1.565$, and it gets the maximum value 0.992 here. Physically, as the nonextensive parameter increases in the $q_{F_\mathrm{i}}\in (3/5, 1.565)$ interval, the closer the determination coefficient is to 1, the closer the fitting result is to the reality; as the nonextensive parameter increases in the interval of $q_{F_\mathrm{i}}\in (1.565, 2)$, the coefficient of determination gradually moves away from 1, and the fitting result is less true. Coefficient of determination can be negative, and when it is negative, the fitting effect is poor^51^. The results are the same as the results of the goodness of fit method measured by *SSE*, which confirms the reliability of our fitting results.
